# Deciphering the role of per- and polyfluoroalkyl substances in prostate cancer: a multi-omics and computational toxicology approach

**DOI:** 10.3389/fcell.2026.1786248

**Published:** 2026-06-01

**Authors:** Kuiyuan Zhang, Bangwei Che, Wei Li, Heng Luo

**Affiliations:** 1 Department of Urology, First Affiliated Hospital of Guizhou University of Traditional Chinese Medicine, Guiyang, China; 2 State Key Laboratory of Discovery and Utilization of Functional Components in Traditional Chinese Medicine, Guizhou Medical University, Guiyang, China

**Keywords:** machine learning framework, multi-omics analysis, network toxicology, per- and polyfluoroalkyl substances, prostate cancer

## Abstract

**Background:**

Per- and polyfluoroalkyl substances (PFAS), persistent environmental contaminants, are associated with increased Prostate cancer (PCa) risk. However, their molecular mechanisms are poorly defined.

**Methods:**

We employed a comprehensive computational and experimental framework. The toxicological profiles of PFOA and PFOS were predicted. Shared molecular targets between PFAS and PCa were identified by integrating toxicogenomic and transcriptomic data, followed by protein-protein interaction network and enrichment analyses. A robust prognostic model was built and validated using multiple machine-learning algorithms. Core targets were further investigated via single-cell/spatial transcriptomics and molecular docking. Key findings were functionally validated in DU145 PCa cells using qPCR, Western blotting, and assays for proliferation, migration, and invasion.

**Results:**

Computational analysis confirmed the carcinogenic and endocrine-disrupting potential of PFAS. We identified 219 common targets significantly enriched in inflammation, oxidative stress, and cancer-related pathways like PPAR and p53 signaling. Network topology highlighted key hub genes, including ALB and PPARG. A 10-gene machine-learning model demonstrated strong prognostic performance (average C-index: 0.710). Cross-omics analyses pinpointed CDC20 as a pivotal core gene within the PFAS-PCa network. Molecular docking indicated stable binding of PFAS to core targets like CDC20. *In vitro* experiments confirmed that PFAS exposure upregulates CDC20 and enhances the malignant phenotypes of PCa cells. Furthermore, docking suggested several natural compounds (e.g., quercetin) could potentially bind CDC20 to mitigate PFAS effects.

**Conclusion:**

This work systematically reveals that PFAS exposure is associated with PCa progression, potentially involving dysregulation of core genes such as CDC20 and perturbing critical cancer pathways. The developed prognostic model holds clinical relevance, and the identified natural products offer a foundation for designing interventions to potentially mitigate PFAS-associated carcinogenic effects, advancing both mechanistic understanding and preventive strategies. However, the findings are primarily based on computational predictions and a single cell line; further validation in multiple models and experimental systems is required.

## Background

Prostate cancer (PCa), the most common cancer among men globally and the second leading cause of cancer-related mortality in males, has seen a significant rise in incidence in recent decades. Global projections estimate over one million new cases and 400,000 deaths annually by 2026, posing a significant public health threat and imposing a substantial healthcare burden (Wang et al., 2018; Kratzer et al., 2025; Zhang Y. et al., 2025). However, the exact underlying mechanisms for its high incidence remain unclear.

The development and progression of PCa involve complex interactions among genetic susceptibility, environmental toxin exposure, and metabolic dysregulation (Magnifico et al., 2025; Constâncio et al., 2025). Among environmental factors, per- and polyfluoroalkyl substances (PFAS), a class of synthetic chemicals widely used in industrial and consumer products such as non-stick cookware, waterproof fabrics, and food packaging, have emerged as a major public health concern due to their environmental persistence and bioaccumulative potential (Zheng et al., 2024; Botelho et al., 2025; Steenland and Winquist, 2021). Notably, a growing body of epidemiological and preclinical evidence suggests an association between PFAS exposure and PCa risk: several studies report elevated PFAS levels in PCa patients and propose that PFAS have been hypothesized to influence carcinogenic processes through hormonal disruption and interference with cellular functions (Troeschel et al., 2024; Yang et al., 2025; Hu et al., 2022; Qian et al., 2025). Despite these findings, current research has limitations: (1) animal studies often employ high-dose PFAS exposure models, which may not accurately reflect real-world human exposure levels; and (2) The specific molecular mechanisms linking PFAS to PCa remain poorly defined.

**FIGURE 1:**
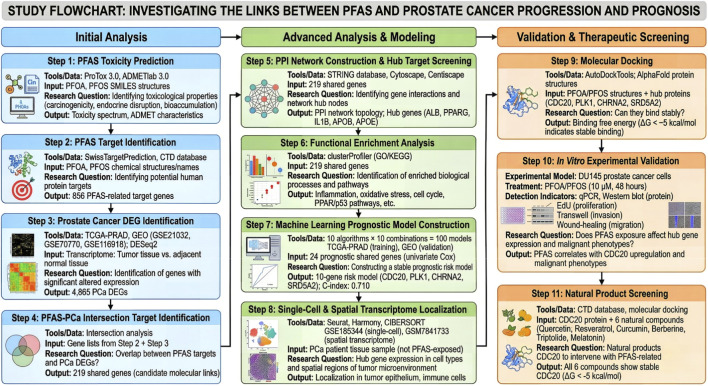
Overall flowchart of this study.

Compared with previous studies that mainly focused on epidemiological associations or single-gene analyses, our study adopts a multi-level integrated strategy that combines network toxicology, multi-omics data (transcriptomics, single-cell and spatial transcriptomics), machine learning, molecular docking, and *in vitro* functional validation. This approach differs from conventional bioinformatic studies by (i) systematically linking PFAS exposure to PCa prognosis via a robustly validated 10-gene risk model; (ii) integrating single-cell and spatial transcriptomics to localize core gene expression in the tumor microenvironment; and (iii) providing both computational predictions and experimental validation of CDC20 as a key mediator. To our knowledge, such a comprehensive framework has not been previously applied to investigate PFAS-associated prostate cancer. The detailed process can be seen in Figure 1.

## Methods and materials

### Data sources

This analysis incorporated transcriptomic profiles and corresponding clinical records from 1,028 prostate cancer patients, derived from two primary sources: the TCGA-PRAD cohort (retrieved from the UCSC Xena platform) and three independent Gene Expression Omnibus (GEO) datasets (GSE21032, GSE70770, GSE116918). To bolster statistical robustness, samples from GSE21032 and GSE70770 were pooled into a unified cohort, with non-biological batch effects mitigated using the ComBat algorithm within the sva R package. Inclusion criteria required available recurrence data and a minimum follow-up period of 1 month. Cohort-specific demographic and clinical details are provided in Supplementary Tables S1,S2. Additionally, a single-cell RNA-seq dataset (GSE185344) and one spatial transcriptomics sample (GSM7841733) from a PCa patient were downloaded from GEO. These single-cell and spatial transcriptomic data were derived from prostate cancer patients (without specific PFAS exposure annotation) and were used to localize the expression of core PFAS-PCa genes within the tumor microenvironment, complementing the bulk transcriptomic DESeq analysis.

### Toxicity prediction

Given their widespread environmental presence and substantial relevance to human exposure risks (Hong et al., 2025a), perfluorooctanoic acid (PFOA) and perfluorooctanesulfonic acid (PFOS) were chosen as representative PFAS for analysis. Their canonical SMILES notations, retrieved from the PubChem database, served as input for a comprehensive toxicity evaluation using the ProTox 3.0 and ADMETlab 3.0 computational platforms. These integrated tools assess a wide spectrum of toxicological endpoints, providing a detailed profile of absorption, distribution, metabolism, excretion, and toxicity (ADMET) characteristics. This computational profiling offers a foundational basis for guiding subsequent experimental investigations and supporting informed risk assessment.

### Identification of PFAS targets

The chemical structures and SMILES representations of PFOA and PFOS were acquired from PubChem. These were processed through the SwissTargetPrediction tool to forecast putative human protein interactions, applying a filter for *Homo sapiens* targets with a non-zero probability score. In parallel, scientifically documented molecular targets were extracted from the Comparative Toxicogenomics Database (CTD) by querying the compound names, limiting results to entries with direct experimental evidence. The target lists from these two sources were subsequently merged. Redundancies were eliminated and gene nomenclature was unified using the UniProt database for consistency.

### Identification of PCa targets

Transcriptome profiling was performed on 534 samples from the TCGA-PRAD cohort. The comparison group consisted of 497 prostate adenocarcinoma tumor tissues and 37 adjacent normal prostate tissues. All tumor samples were primary tumors obtained from surgical resection without neoadjuvant therapy, and the normal tissues were paired non-malignant prostate samples. This comparison aimed to identify transcriptomic alterations (DEGs) in tumors relative to normal tissues. Using the DESeq2 R package, differentially expressed genes were identified based on thresholds of |log_2_ fold change| > 0.65 and an adjusted p-value < 0.05. The resulting gene set, representing significant expression alterations in tumor tissues, was used as a candidate pool for subsequent integrative analysis with PFAS-associated targets. These overlapping genes should be interpreted as potential molecular links warranting experimental validation, rather than evidence of direct causal relationships.

### Identification of PFAS-PCa targets and construction of protein-protein interaction (PPI) network

Shared molecular targets between PFAS exposure and prostate cancer were identified through intersection analysis. This overlap was used as a hypothesis-generating step to prioritize candidate genes for subsequent functional investigation, rather than to infer causality. An interaction map of these shared targets was subsequently built via the STRING database. The network was visualized in Cytoscape (version 3.10.3). Key topological metrics, degree, closeness, and betweenness centrality, were computed using the Centiscape 2.0 plug-in. Nodes were prioritized according to their closeness centrality scores, reflecting their positional influence in the network.

### Enrichment analysis

Functional enrichment analysis of the PFAS-PCa targets was performed using the R package “clusterProfiler” for Gene Ontology (GO) and Kyoto Encyclopedia of Genes and Genomes (KEGG) pathway analysis, linking genomic information to functional pathways at the systems and molecular levels. Finally, only results with an adjusted p-value less than 0.05 were considered statistically significant and visualized using the R package “ggplot2.”

### Development and validation of a PFAS-PCa risk model

First, we performed univariate COX regression analysis to screen for 219 PFAS-PCa overlapping genes that were consistently associated with PCa prognosis in the TCGA-PRAD and GSE cohorts. Subsequently, based on these genes and an established analytical pipeline (Li et al., 2025; Zhang K. et al., 2025), we constructed a prognostic modeling framework: by evaluating 100 different combinations derived from 10 machine learning algorithms, using the TCGA-PRAD cohort as the training set and the GSE and GSE116918 cohorts as the test sets. The model combinations were compared according to the average concordance index (C-index), and the best-performing combination was selected as the final model to pinpoint core genes involved in the disease pathological process. Next, we applied this final model to calculate the risk score for each patient; within each cohort, the median risk score was used as the cutoff, with patients scoring above the median classified as high-risk group and those scoring below or equal to the median as low-risk group, thereby ensuring consistent grouping criteria across cohorts. Finally, we evaluated survival differences between these subgroups using Kaplan-Meier analysis, and quantified the discriminative ability of the model by calculating the area under the ROC curve (AUC).

### Single-cell and spatial transcriptomic analysis

In the single-cell RNA-seq processing workflow, rigorous quality filtering was implemented: cells were retained only if they exhibited mitochondrial gene content under 20%, expressed over 200 genes, and were detected in a minimum of 3 cells, yielding 36,424 qualified cells for downstream analysis. Batch effects were harmonized using the Harmony algorithm. Following log-normalization, the top 2000 highly variable genes were selected via the FindVariableFeatures function. Dimensionality reduction was conducted with principal component analysis, after which soft k-means clustering was carried out through the Harmony package; final cell clusters were determined with the FindClusters function at a resolution of 0.3. Cell identities were assigned according to established marker genes, differential expression profiles, and recognized lineage attributes. Spatial transcriptomic datasets were similarly processed, normalized, and standardized with the Seurat package, and tissue regions were annotated based on classic marker genes. Examination of the expression patterns of key PFAS-PCa-associated targets across distinct cell types helped clarify potential cellular mechanisms connecting PFAS exposure to prostate cancer advancement. Subsequently, the CIBERSORT algorithm was employed to assess the influence of the core gene CDC20 on the tumor microenvironment composition.

### Molecular docking

To investigate the interaction mechanisms between PFAS and core PFAS-PCa target proteins, computational docking analysis was conducted. The molecular structures of PFOA and PFOS were sourced from the PubChem database, and the three-dimensional conformations of the target proteins were retrieved from the AlphaFold Protein Structure Database. AutoDockTools 1.5.7 was used for structural preparation of the ligands and receptors and to perform docking simulations. These simulations aimed to predict binding modes, binding affinity (quantified as binding free energy, ΔG), and potential functional implications. A binding free energy below 0 kcal/mol indicates spontaneous binding, while a value lower than −5.0 kcal/mol suggests the formation of a stable complex with strong binding capability. To ensure reliability, the docking energy for PFAS interactions with core PFAS-PCa target proteins was calculated as the mean value from three independent simulation experiments.

### Cell culture and treatment

The human prostate cancer cell line DU145 was obtained from the Cell Bank of the Chinese Academy of Sciences. Cells were maintained at 37 °C in a 5% CO_2_ atmosphere using culture conditions specified by the supplier. For perfluorooctanoic acid (PFOA) and perfluorooctanesulfonic acid (PFOS) exposure, previously established concentration protocols were followed (Gałęzowska et al., 2026; Charazac et al., 2022). DU145 cells were treated with 10 nM PFOA or PFOS for 48 h. To investigate whether CDC20 is required for PFAS-induced malignant phenotypes, small interfering RNA (siRNA) targeting CDC20 (si-CDC20). DU145 cells were seeded in six-well plates at 50%–60% confluence and transfected with 50 nM si-CDC20 using Lipofectamine 3000 (Invitrogen, United States) according to the manufacturer’s protocol. After 24 h of transfection, cells were treated with 10 nM PFOA or PFOS for an additional 48 h. Knockdown efficiency was confirmed by qPCR.

### RNA isolation and quantitative real-time PCR (qPCR)

Gene expression levels were quantified using established molecular biology protocols. Total RNA was isolated with TRIzol reagent, and its concentration and integrity were verified using a NanoDrop 2000 spectrophotometer. Subsequently, high-quality RNA was reverse-transcribed into complementary DNA (cDNA) employing the PrimeScript™ RT Reagent Kit. Quantitative real-time PCR (qPCR) was then carried out on a QuantStudio 5 platform with the Premix Ex Taq™ Kit, under the following thermal cycling conditions: an initial denaturation at 95 °C for 30 s, followed by 40 cycles of 95 °C for 5 s and 60 °C for 34 s. All assays included three technical replicates. The specific primer sequences utilized in this study are provided in Supplementary Table S3.

### Western blotting

Protein extraction from PFAS-exposed and control DU145 cells was carried out using RIPA lysis buffer (Solarbio, China) containing a protease inhibitor mixture (Yeasen, China). The lysates were resolved on 10% SDS-polyacrylamide gels and subsequently transferred to PVDF membranes via a semi-dry transfer system. After blocking with 5% non-fat milk for 1 hour at ambient temperature, the membranes were probed with a primary antibody against CDC20 (Proteintech, Cat# 10252-1-AP) at a 1:7,000 dilution overnight at 4 °C. Following TBST washes, incubation with an appropriate horseradish peroxidase-conjugated secondary antibody was performed for 2 hours at room temperature.

### EdU proliferation assay

An EdU-based proliferation assay (Beyotime Biotechnology, Shanghai) was used to evaluate the proliferative response of DU145 cells following PFOS and PFOA exposure. Cells, either treated or untreated, were plated in 24-well plates at 3 × 10^3^ cells per well. Following a 24-h incubation, 20 µL of EdU solution was introduced to each well and the cells were further cultured for 2 h at 37 °C. Subsequent fixation and staining steps were conducted as per the kit protocol. EdU-positive nuclei were finally visualized and imaged using a fluorescence microscope at ×20 magnification.

### Wound healing assay

To assess the migratory capacity of DU145 cells following PFOA or PFOS treatment, cells were plated into six-well dishes and grown to complete monolayer coverage. A linear wound was introduced using a sterile 200 µL pipette tip. After rinsing with phosphate-buffered saline to clear dislodged cells, a consistent *in vitro* wound model was generated. To reduce potential confounding by cell proliferation, subsequent culture was performed in medium containing low serum. Wound closure was monitored and imaged immediately after scratching (0 h) and again at 48 h using phase-contrast microscopy (×10 objective).

### Transwell invasion assay

A suspension of cells in serum-free medium was placed in the upper compartment of a Transwell chamber (8 μm pore polycarbonate membrane). The lower compartment contained RPMI-1640 medium supplemented with 10% FBS to serve as a chemoattractant. Following 24 h of incubation at 37 °C under 5% CO_2_, cells that had traversed the membrane were fixed using 4% paraformaldehyde and stained with 0.1% crystal violet. For quantification, five randomly selected fields per membrane were imaged with an inverted microscope (×10 objective), and migrated cells were enumerated.

### Screening of natural active products

To explore natural compounds that may mitigate the detrimental impacts of endocrine-disrupting chemicals, this investigation focused on CDC20, a central hub within the PFAS-PCa network. Candidate substances associated with CDC20 were retrieved from the Comparative Toxicogenomics Database and subsequently evaluated for their binding affinity using computational docking approaches.

## Results

### Preliminary analysis of the PFAS toxicity network

Computational toxicity profiling was conducted using ADMETlab 3.0 and ProTox 3.0 to evaluate PFOA and PFOS. Both compounds demonstrated marked carcinogenicity and a propensity for bioaccumulation. Pharmacokinetic predictions indicated efficient gastrointestinal uptake (Caco-2 Papp > 1.2 × 10^−6^ cm/s), high lipophilicity (LogP > 5), and slow systemic clearance (plasma CL < 10 mL/min/kg), supporting their potential for long-term retention in biological systems. Toxicity endpoints further confirmed carcinogenic and mutagenic hazards. Additionally, these substances were predicted to interact with several receptors—including the aryl hydrocarbon receptor, estrogen/androgen receptors, aromatase, and thyroid hormone receptors, suggesting broad endocrine-disrupting capability. These *in silico* results lend theoretical support to the notion that PFAS may contribute to prostate cancer progression through metabolic interference and receptor modulation, thereby informing subsequent mechanistic research.

### Identification of PFAS-PCa targets

**FIGURE 2:**
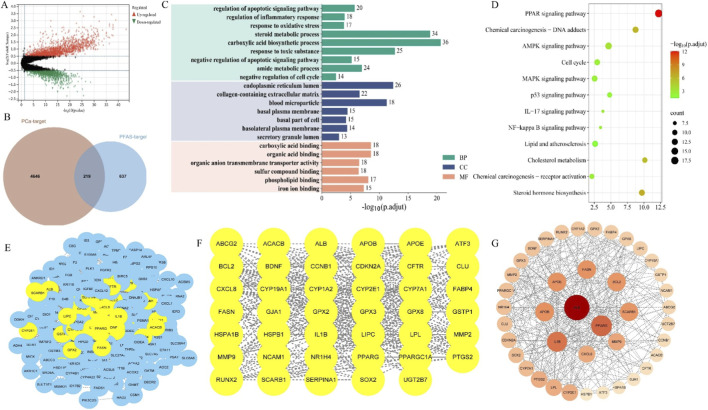
Identification, enrichment analysis, and PPI network construction of PFAS-PCa Targets. **(A)** Volcanic plot of differentially expressed genes between normal and tumor groups in prostate cancer; **(B)** PFAS-PCA overlapping gene; **(C)** GO enrichment analysis; **(D)** KEGG enrichment analysis; **(E–G)** Construction of PPI network and screening of core targets.

Following the consolidation of target predictions from the SwissTargetPrediction and CTD, 856 distinct genes associated with PFAS were compiled (Supplementary Table S4). Concurrently, analysis of expression differences between tumor and adjacent normal tissues revealed 4,865 genes with altered expression levels (tumor vs. normal) (Supplementary Table S4). Intersection analysis highlighted 219 genes common to both PFAS exposure and prostate cancer (Figure 2A,B). These shared genes represent candidate molecular targets that may link PFAS exposure to PCa-related processes, providing a basis for hypothesis generation and subsequent experimental exploration. The identification of these overlapping genes does not imply causality but rather serves to prioritize targets for further functional validation.

### Enrichment analysis of PFAS-PCa targets

Functional enrichment analysis based on GO revealed that the 219 PFAS–PCa-associated genes were prominently enriched in biological processes related to inflammatory and oxidative stress responses, cellular proliferation, apoptosis, and metabolic reprogramming (Figure 2C). KEGG pathway analysis further demonstrated the involvement of these genes in several key signaling and disease-relevant pathways, including PPAR signaling, steroid hormone biosynthesis, MAPK signaling, cell cycle and cellular senescence, p53 signaling, chemical carcinogenesis, as well as transcriptional dysregulation in cancer (Figure 2D). These enrichment results suggest that PFAS may be involved in PCa-related processes, including inflammatory and oxidative stress pathways, cell proliferation, and cancer-associated signaling.

### Construction of the PPI network for PFAS-PCa targets

The 219 shared genes linking PFAS exposure to PCa were subjected to PPI analysis using the STRING database, with an interaction confidence cutoff set at ≥0.4. Following the removal of unconnected nodes, a network comprising 197 proteins was obtained and visualized in Cytoscape 3.10.3. In the resulting PPI map, nodes were sized and colored according to their degree centrality, where larger and darker nodes represent higher connectivity within the network. Topological evaluation highlighted five central hub genes, ALB, PPARG, IL1B, APOB, and APOE, as key players in the PFAS-PCa interactome (Figures 2E–G). This network representation not only delineates functional associations among critical targets but also offers mechanistic insights into how PFAS may contribute to prostate cancer pathogenesis.

### Development and evaluation of the PFAS-PCa prediction model

**FIGURE 3:**
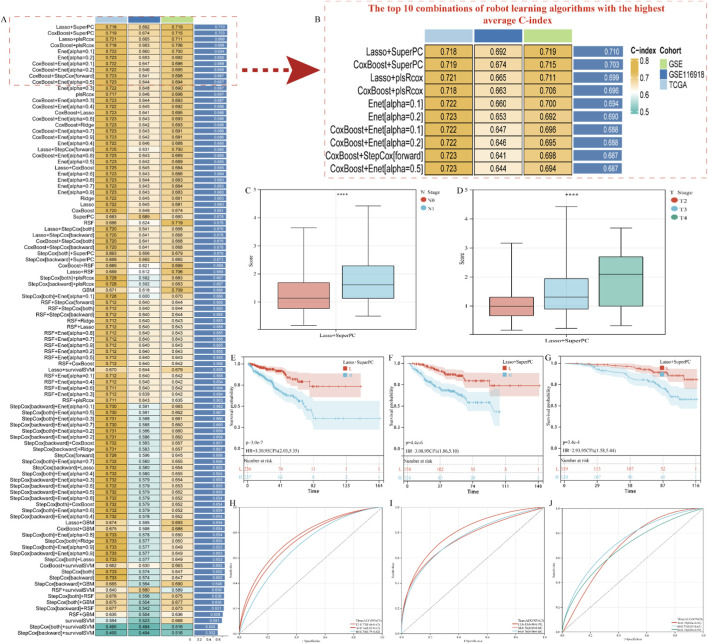
Establishment and validation of PFAS-PCa target related risk prediction model. **(A, B)** The average C-index of 101 machine learning algorithm combinations; **(C, D)** risk score stratified by clinical stage; **(E–G)** comparing survival outcomes using the risk score as a stratifier, **(E)** TCGA-PRAD, **(F)** GSE, GSE116918. **(H–J)** Performance of the risk score in identifying BCR patients by ROC analysis, **(H)** TCGA-PRAD, **(I)** combined GEO cohort, **(J)** GSE116918.

Ten machine learning algorithms were systematically applied to evaluate the 24 PFAS-PCa genes identified by univariate Cox regression analysis (Supplementary Figure S1) as significantly associated with disease-free survival (DFS) in both the TCGA-PRAD and combined GEO cohorts. The Lasso + SuperPC hybrid model was determined as the optimal predictive model. This model integrated ten key genes (Figures 3A,B; Supplementary Table S5) and achieved an average C-index of 0.710. Clinicopathological analysis revealed that the model score was significantly correlated with higher pathological stage (Figures 3C,D). Kaplan-Meier analysis further confirmed that high-risk patients exhibited worse clinical outcomes in three independent cohorts: TCGA-PRAD (Figure 3E), the combined GEO cohort (Figure 3F), and GSE116918 (Figure 3G). Time-dependent ROC curves further validated the model’s predictive accuracy for disease progression at different time points. In the TCGA-PRAD cohort, the AUC values were 0.77 at 1 year, 0.74 at 3 years, and 0.70 at 5 years (Figure 3H). In the combined GEO cohort, the corresponding AUCs were 0.82, 0.76, and 0.76 (Figure 3I). In the GSE116918 cohort, AUCs reached 0.70 at 3 years, 0.74 at 5 years, and 0.70 at 8 years (Figure 3J). Collectively, these results demonstrate the substantial prognostic value of the 10-gene PFAS-PCa risk model for prostate cancer outcome prediction.

### Survival analysis of component genes in the PFAS-PCa risk model

Kaplan-Meier survival analysis of the 10-gene PFAS-PCa risk model identified four genes whose expression was consistently and significantly associated with disease-free survival across all three validation cohorts (Supplementary Figure S2). Among these, elevated expression of CDC20 and PLK1 correlated with shorter DFS, indicating a risk-promoting role. In contrast, higher levels of CHRNA2 and SRD5A2 were linked to longer DFS, suggesting a protective function. Given their robust and reproducible prognostic performance, these four genes were designated as core candidates for subsequent mechanistic studies exploring how PFAS exposure influences prostate cancer development and progression.

### The impact of PFAS on core PFAS-PCa targets

**FIGURE 4:**
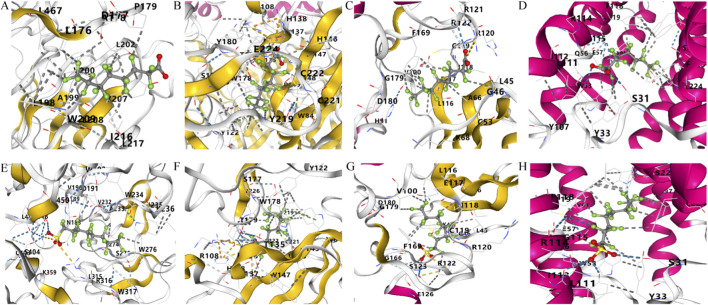
Binding of PFAS to core target proteins, **(A)** PFOA-CDC20, **(B)** POFA-CHRNA2, **(C)** PFOA- PLK1, **(D)** PFOA-SRD5A2, **(E)** PFOS-CDC20, **(F)** PFOS- CHRNA2, **(G)** POFS- PLK1, **(H)** POFS- SRD5A2.

Molecular docking simulations were conducted to assess potential interactions between PFAS (PFOA and PFOS) and the four core protein targets. The results demonstrated that both compounds could form stable complexes with all four proteins, with binding free energies (ΔG) consistently below −5 kcal/mol, indicating strong binding affinity. For PFOA, the calculated ΔG values were: CDC20 (−7.5 kcal/mol, Figure 4A), CHRNA2 (−10.1 kcal/mol, Figure 4B), PLK1 (−8.1 kcal/mol, Figure 4C), and SRD5A2 (−9.0 kcal/mol, Figure 4D). Similarly, PFOS exhibited stable binding to CDC20 (−8.0 kcal/mol, Figure 4E), CHRNA2 (−10.2 kcal/mol, Figure 4F), PLK1 (−8.5 kcal/mol, Figure 4G), and SRD5A2 (−10.2 kcal/mol, Figure 4H). These computational findings suggest that PFOA and PFOS can directly and stably interact with the four core proteins, supporting their potential functional role in PFAS-mediated prostate cancer initiation and progression.

### Expression localization and validation of core genes

**FIGURE 5:**
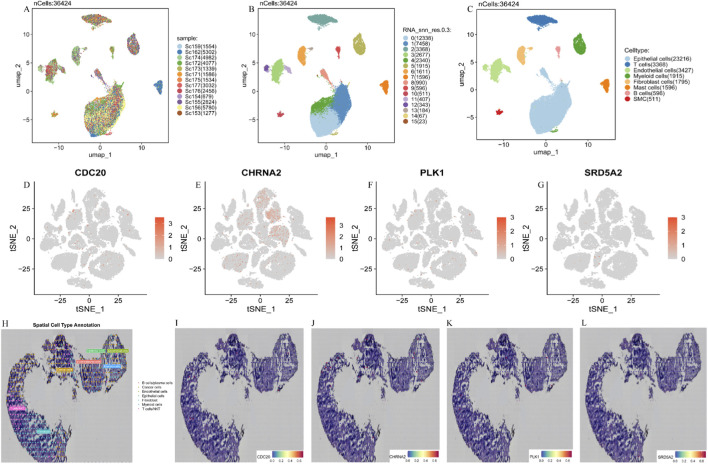
The expression of core genes in microenvironmental cells. **(A)** single cell clustering plot by sample group; **(B)** single cell clustering plot by cell cluster group; **(C)** single cell clustering plot of eight microenvironment cell populations; **(D–G)** expression localization of core genes in single cell clusters; **(H)** Patial map of tumor microenvironment cellular composition on tissue sections; **(I–L)** Spatial cellular mapping of core genes in the tumor microenvironment.

**FIGURE 6:**
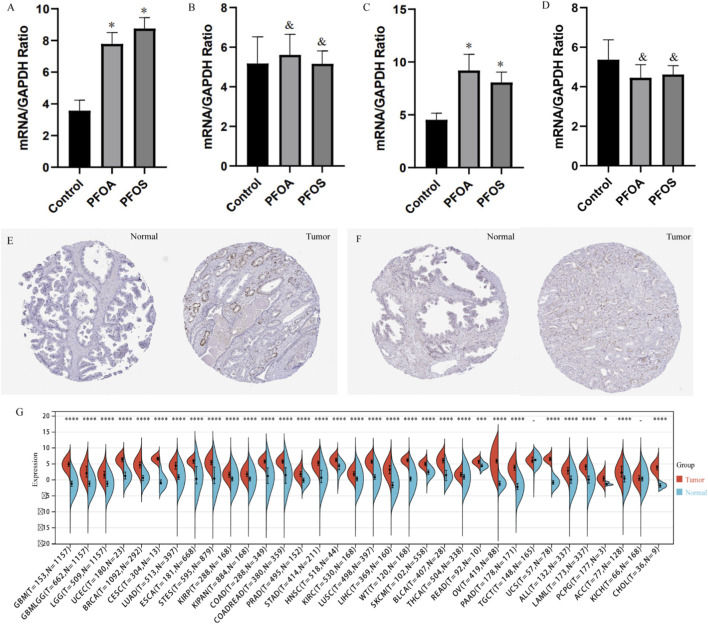
Verification of core gene expression. **(A–D)** qPCR analysis of mRNA expression in DU145 cells treated with 10 nM PFOA or PFOS for 48 h: **(A)** CDC20, **(B)** CHRNA2, **(C)** PLK1, **(D)** SRD5A2. Data are mean ± SD. *p < 0.05 vs. control; ^&^p < 0.05 vs. control. **(E, F)** Protein expression validation using the Human Protein Atlas (HPA) database in normal prostate and prostate cancer tissues (without PFAS treatment): **(E)** CDC20, **(F)** PLK1. **(G)** Pan-cancer analysis of CDC20 expression across multiple cancer types (SangerBox database). Note: panels **(E, F)** show baseline protein expression in clinical tissue samples and are not derived from PFAS-exposed systems.

Following Harmony-based batch correction, cellular distributions across samples became well-integrated, indicating effective mitigation of technical batch effects (Figure 5A). At a clustering resolution of 0.3, 17 distinct subclusters were identified (Figure 5B), which were subsequently annotated into seven major cell types using canonical marker genes (Supplementary Figure S3): epithelial cells, T cells, B cells, macrophages, endothelial cells, mast cells, and smooth muscle cells (Figure 5C). Expression analysis demonstrated that the four core genes were widely distributed across these diverse cell populations (Figures 5D–G). Spatial transcriptomics further highlighted their pronounced expression, particularly within tumor regions (Figures 5H–L). qPCR assays confirmed that PFAS exposure selectively elevated mRNA levels of CDC20 and PLK1 (Figures 6A–D). Protein-level validation using the Human Protein Atlas (HPA) database revealed upregulated CDC20 expression in prostate cancer tissues, whereas PLK1 showed moderate expression in both tumor and adjacent normal tissue (Figures 6E,F; Supplementary Table S6). Based on its consistent prognostic significance across cohorts, PFAS-induced transcriptional upregulation, and corroborated protein overexpression, CDC20 was selected as the principal target for subsequent mechanistic investigation.

### Pan-cancer analysis of the core gene *CDC20* and its relationship with the microenvironment

Pan-cancer analysis (based on the SangerBox database) revealed that *CDC20* was significantly upregulated in almost all cancer types (Figure 6G). Its high expression was significantly associated with disease-specific survival in multiple tumors, including GBMLGG, KIPAN, KIRP, KIRC, LGG, ACC, KICH, MESO, LIHC, PAAD, LUAD, SKCM, PRAD, SKCM-M, SARC, THCA, and BRCA (Supplementary Figure S4). These results collectively underscore the important role of *CDC20* in tumorigenesis and development, suggesting it may be a key molecular target in PFAS-driven carcinogenesis. Subsequently, immune infiltration analysis showed that the *CDC20* high-expression group exhibited higher infiltration levels of immunosuppressive cells (Tregs and M2 macrophages) (Supplementary Figure S4A). Correlation analysis further confirmed a positive association between *CDC20* expression levels and the degree of immunosuppressive cell infiltration (Supplementary Figures S4B,C).

### Effects of PFAS on PCa cells *in vitro*

**FIGURE 7:**
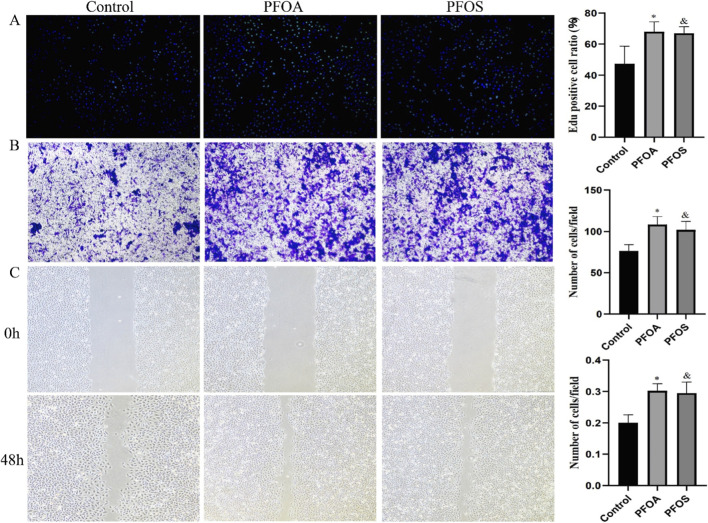
The effect of PFAS on prostate cancer cells (DU145). **(A)** EdU proliferation assay experiment; **(B)** Invasion experiment; **(C)** Cell scratch test. Note: *p < 0.05, PFOA vs. Control; ^&^p < 0.05, PFOS vs. Control.

Western blot analysis showed that PFAS treatment significantly increased CDC20 protein expression levels in PCa cells (Supplementary Figure S4D). Further cell functional assays demonstrated that PFAS intervention significantly promoted the proliferation (EdU assay, Figure 7A), invasion (Transwell assay, Figure 7B), and migration (wound healing assay, Figure 7C) of PCa cells. These results further emphasize that PFAS may promote the malignant progression of PCa by upregulating *CDC20* expression. To determine whether CDC20 is functionally required for these PFAS-induced effects, we knocked down CDC20 using specific siRNA in PFAS-treated DU145 cells. Remarkably, CDC20 knockdown substantially reversed the PFAS-promoted malignant phenotypes: the enhanced proliferation, migration, and invasion capacities observed upon PFOA or PFOS exposure were all significantly attenuated in si-CDC20-transfected cells (Supplementary Figures S5A–C). These results indicate that CDC20 plays a critical and necessary role in mediating the pro-cancer effects of PFAS in prostate cancer cells.

### Screening of potential interventional natural active products

**FIGURE 8:**
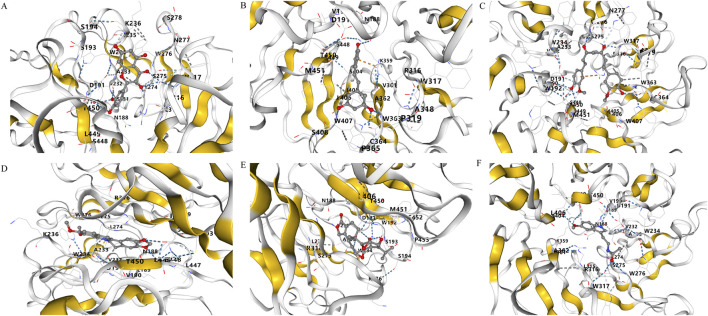
Binding of 6 Natural Active Products to CDC20 Protein. **(A)** quercetin, **(B)** resveratrol, **(C)** curcumin, **(D)** berberine, **(E)** triptolide, **(F)** melatonin.

Recent experimental evidence increasingly suggests that natural active products hold significant potential in PCa drug development due to their multi-target and multi-pathway characteristics. To explore potential compounds that might mitigate the adverse effects of PFAS exposure, this study searched the CTD for natural active components related to *CDC20*, identifying six natural products associated with *CDC20*: quercetin, resveratrol, curcumin, berberine, triptolide, and melatonin. Further molecular docking analysis indicated that all six natural active components could bind stably to CDC20 (ΔG < −5 kcal/mol): quercetin (−8.2 kcal/mol) (Figure 8A), resveratrol (−6.8 kcal/mol) (Figure 8B), curcumin (−7.6 kcal/mol) (Figure 8C), berberine (−8.2 kcal/mol) (Figure 8D), triptolide (−8.5 kcal/mol) (Figure 8E), and melatonin (−6.5 kcal/mol) (Figure 8F). These computational findings are hypothesis-generating and provide a rationale for future experimental testing of these natural products as potential modulators of CDC20. We emphasize that docking predictions alone do not demonstrate biological activity or therapeutic efficacy, and subsequent *in vitro* and *in vivo* studies are required to validate any functional effects.

## Discussion

As exogenous environmental contaminants, PFAS can disrupt physiological homeostasis by interfering with the endocrine and metabolic systems, exerting multiple adverse effects on the reproductive, nervous, and immune systems. With industrial development, PFAS have become widespread environmental pollutants. Long-term, multi-pathway human exposure to these substances may pose significant health risks, particularly related to their carcinogenic properties (Zheng et al., 2024; Botelho et al., 2025; Steenland and Winquist, 2021). Multiple lines of laboratory evidence indicate a clear association between PFAS exposure and an increased risk of PCa: an epidemiological study involving 1,610 participants showed that high PFOS levels were associated with elevated PCa risk (Troeschel et al., 2024); a recent meta-analysis reached a similar conclusion (Yang et al., 2025). *In vitro* experiments have further confirmed that PFAS can promote the proliferation and invasion of prostate cancer cells (Hu et al., 2022; Qian et al., 2025). These studies, from population data to the cellular level, collectively support a strong association between PFAS exposure and increased PCa risk.

Although existing research has established an association between PFAS exposure and PCa risk (Troeschel et al., 2024; Yang et al., 2025; Hu et al., 2022; Qian et al., 2025; Imir et al., 2021; Hong et al., 2025b; Boyd et al., 2022; Moon and Mun, 2024), several key scientific questions remain unresolved. First, most current studies focus on epidemiological correlation analyses, with insufficient exploration of the specific molecular targets and mechanisms by which PFAS may influence PCa initiation and progression. Second, traditional experiments are often limited to detecting changes in specific protein expression, lacking systematic analysis of overall biological processes and interaction networks. High-throughput targeted screening methods offer a more comprehensive way to reveal molecular alterations induced by PFAS. Third, the prognostic value of PFAS-related molecular markers in PCa patients remains unclear, limiting their clinical translational potential. Furthermore, given the high heterogeneity of the tumor microenvironment (TME), the impact of PFAS on non-malignant cells (e.g., immune cells, stromal cells) and their role in PCa progression remain largely unknown. These research gaps highlight the necessity of employing a multi-omics integration strategy to systematically elucidate the mechanisms underlying the PFAS-PCa association.

To address these gaps, we implemented a multidisciplinary strategy based on multi-omics data, integrating bioinformatics, machine learning, molecular docking, and *in vitro* assays. This approach allowed us to systematically investigate the PFAS-PCa relationship, identify key genes and regulatory networks, and screen for natural bioactive compounds that might mitigate the PFAS exposure-associated increase in PCa risk. In this study, we selected the two most common PFAS, PFOA and PFOS, based on their environmental prevalence and significant potential for human carcinogenic exposure. Subsequent toxicity assessment confirmed their bioaccumulation tendency, carcinogenic potential, metabolic effects, and endocrine-disrupting activities. Our bioinformatic analysis identified 219 bridging genes potentially linking PFAS exposure to PCa pathogenesis. KEGG and GO enrichment analyses suggested that PFAS may be involved in PCa-related biological processes, including inflammatory and oxidative stress responses, cell proliferation, and metabolic reprogramming. This appears to occur through disruption of key cancer-related signaling pathways, including TP53, PPAR, and AMPK. These findings underscore the multifaceted impact of PFAS on PCa, potentially involving multiple targets and pathways. While these pathways are broadly cancer-relevant, their modulation by PFAS exposure in the context of PCa requires further experimental testing. Furthermore, using a robust computational framework involving 100 combinations of 10 machine learning algorithms and subsequent survival analysis, we identified CDC20, CHRNA2, PLK1, and SRD5A2 as core targets in the PFAS-PCa network, as their expression levels were significantly correlated with patient survival across multiple cohorts. Integrating single-cell RNA sequencing, spatial transcriptomics, and immune infiltration analysis revealed the specific expression patterns of these core genes within the TME. Notably, CDC20—whose mRNA and protein levels were confirmed to be regulated by PFAS in vitro—correlated with the infiltration degree of immunosuppressive cells (Tregs and M2 macrophages). This provides new insights into the mechanism whereby PFAS not only affects malignant cells but also modulates stromal and immune cell populations.

Of particular note, the four core targets play distinct roles in PCa. Multiple studies have shown that CDC20 plays a critical role in PCa pathogenesis (Wei et al., 2020; Luo et al., 2020; Wu et al., 2017; Wu et al., 2018; Wood et al., 1995). As an oncogene located in the frequently amplified 9p chromosomal region, CDC20 cooperates with PLK1 and CDK1 to drive cell cycle progression and metastasis; its overexpression is significantly associated with poor prognosis and increased biochemical recurrence risk (Wei et al., 2020; Luo et al., 2020). Mechanistically, CDC20 is a substrate for SPOP-mediated ubiquitination and degradation (Wu et al., 2017). SPOP mutations in PCa enhance CDC20 protein stability, consequently inducing docetaxel resistance (Wu et al., 2017; Wu et al., 2018). In contrast, the role of CHRNA2 in prostate cancer remains poorly understood. This gene, located at 8p21, encodes a neurotransmitter receptor subunit and has been identified as a downstream target of the androgen receptor signaling pathway (Wood et al., 1995; Fan et al., 2022). Experimental evidence indicates its expression is suppressed by dihydrotestosterone (DHT) but is significantly upregulated in patients receiving androgen deprivation therapy (Fan et al., 2022), suggesting its potential involvement in PCa progression. Similar to CDC20, PLK1, a serine/threonine kinase, plays a central role in regulating cell cycle progression (Parashara et al., 2024; Conti et al., 2024). It participates in multiple key mitotic events, including mitotic entry, centrosome maturation, spindle assembly, sister chromatid cohesion, and cytokinesis (Parashara et al., 2024; Conti et al., 2024), and has been linked to PCa progression, including metastasis, recurrence, and anti-androgen resistance (Tang et al., 2025; Shin et al., 2019; Liang et al., 2023). SRD5A2 is a key enzyme highly expressed in prostate tissue that catalyzes the conversion of testosterone to the more potent DHT (Choubey et al., 2015). Reduced SRD5A2 expression may lead to decreased DHT production, thereby attenuating androgen receptor-dependent gene regulation, affecting cell differentiation, apoptosis, and proliferation, and ultimately disrupting prostate cellular homeostasis while increasing malignant transformation risk (Sharkey et al., 2024; Akalu et al., 1999). Furthermore, diminished SRD5A2 expression may disrupt redox balance, elevating reactive oxygen species levels and inducing DNA damage and genomic instability, thereby accelerating PCa progression (Yang et al., 2020; Ha et al., 2016). Together, these experimental findings highlight the crucial role of SRD5A2 in PCa initiation and development.

Natural active products have become an important source for anti-tumor drug development due to their multi-target, multi-pathway pharmacological properties (Slika et al., 2022). In recent years, their potential in alleviating health damage caused by environmental pollutant exposure has also garnered increasing attention (Watkins et al., 2007; Ghahri et al., 2024). Based on these findings, exploring natural products that can antagonize the carcinogenic effects of PFAS is of great significance for developing corresponding protective strategies. In this study, we screened six natural active components targeting the core gene CDC20 through the CTD and validated their binding ability to CDC20 via molecular docking. This provides experimental evidence for the application of natural products in the prevention and control of PFAS-associated tumors. It is important to note that all docking results presented in this study are computational predictions. While they suggest potential binding interactions between PFAS or natural compounds and CDC20, these findings require experimental confirmation using orthogonal methods such as surface plasmon resonance, isothermal titration calorimetry, or cellular thermal shift assays, as well as functional assays to assess CDC20 activity or downstream signaling.

Compared to previous studies, this research represents a significant methodological advance. Firstly, we employed a multi-level strategy integrating multi-omics data analysis, bioinformatics, machine learning algorithms, molecular docking simulations, and *in vitro* experimental validation, significantly enhancing the systematicity and scientific rigor of the study. Previous research often relied on database-derived epidemiological surveys and did not utilize multi-omics and multi-dimensional methods to reveal the complex interactions between multiple genes and environmental factors in PCa pathogenesis, demonstrating clear limitations of traditional approaches in this field. Secondly, this study not only deeply revealed key core genes in the carcinogenic mechanism of PFAS but also, through reverse network pharmacology, discovered that six natural active products may ameliorate the carcinogenic effects induced by PFAS. These findings hold certain translational potential: on one hand, they can provide a scientific basis for dietary interventions (e.g., recommending intake of specific natural active ingredients) for high-risk exposed populations; on the other hand, they offer candidate substances and theoretical support for developing novel therapeutic strategies for PCa patients.

To strengthen confidence in our findings, several additional experiments are warranted. First, validation of CDC20 as a direct functional mediator of PFAS effects could be achieved through CRISPR-based knockout or pharmacological inhibition in multiple PCa cell lines (e.g., LNCaP, PC-3). Second, animal models of PFAS exposure (e.g., mouse xenograft or transgenic models) would help assess the *in vivo* relevance of CDC20 upregulation and its contribution to tumor progression. Third, orthogonal binding assays such as surface plasmon resonance or isothermal titration calorimetry are needed to confirm the direct interactions predicted by molecular docking. Our results are consistent with previous reports that PFAS promote cancer cell proliferation via oxidative stress and PPAR activation (Zheng et al., 2024; Hu et al., 2022; Qian et al., 2025), and that CDC20 is an oncogene in prostate cancer (Wei et al., 2020; Luo et al., 2020; Wu et al., 2017; Wu et al., 2018). However, some studies have suggested that PFAS effects may vary by cell type and exposure duration (Gałęzowska et al., 2026; Charazac et al., 2022), and the precise role of CDC20 in PFAS-induced endocrine disruption remains to be clarified. Integrating such supporting and conflicting evidence will be essential for refining the mechanistic model.

Several limitations of this study need to be acknowledged. First, target identification was primarily based on predictions from bioinformatics databases. Such methods are susceptible to algorithm bias and confidence thresholds, which may compromise the accuracy and comprehensiveness of the identified PFAS targets. Moreover, the identification of overlapping genes between PFAS targets and differentially expressed genes in PCa was based on computational predictions and transcriptomic correlations. This overlap does not establish causality but rather serves as a hypothesis-generating strategy to prioritize candidates for mechanistic studies.

Second, experimental validation was conducted in only one cell line. It should be noted that PCa is highly heterogeneous, encompassing different phenotypes such as androgen-sensitive (e.g., LNCaP) and castration-resistant (e.g., DU145, PC-3) subtypes. Given the endocrine-disrupting properties of PFAS, their effects on these subtypes may differ substantially. In this study, we selected an androgen-independent cell line (DU145) to evaluate the direct effects of PFAS on malignant phenotypes while avoiding confounding factors related to androgen responsiveness. However, PFAS may still exert differential impacts on hormone-related pathways, including androgen receptor signaling, steroid metabolism, and neuroendocrine differentiation. Future studies should employ a panel of cell lines covering a spectrum of hormonal states (e.g., androgen-sensitive LNCaP, androgen-responsive VCaP, and AR-negative PC-3) to validate the generalizability of our findings. Therefore, readers should interpret our *in vitro* results as evidence generating hypotheses in the context of castration resistance, warranting further validation in more comprehensive models.

Furthermore, the current research relies on *in vitro* cell models, which cannot fully replicate the complex pharmacokinetics of PFAS *in vivo*, including tissue distribution, protein binding, metabolism, and chronic accumulation. Our findings should therefore be interpreted as mechanistic evidence obtained under controlled experimental conditions, and extrapolation to human exposure scenarios should be made with caution. Future studies employing physiologically based pharmacokinetic (PBPK) modeling or animal exposure models are warranted to bridge the gap between *in vitro* observations and human health risk assessment. Finally, although qPCR and Western blot experiments indicated changes in CDC20 expression levels, the specific upstream regulatory mechanisms and downstream signaling pathways involved require further exploration and validation through additional molecular experiments.

## Conclusion

This study systematically explored potential molecular mechanisms linking PFAS exposure to PCa-related processes by integrating multi-omics data and computational toxicology methods. The research found that PFAS, by interfering with key biological processes such as inflammatory responses, the cell cycle, and metabolic reprogramming, correlates with altered expression of core genes including CDC20 and PLK1, which may contribute to malignant phenotypes in PCa cells. A risk model constructed based on machine learning demonstrated prognostic predictive value across multiple cohorts. Furthermore, the study screened multiple natural active products capable of targeting CDC20, providing a new direction for intervention strategies against PFAS-associated PCa. Nevertheless, the study has limitations including reliance on bioinformatic predictions, lack of internal cross-validation for the machine learning model, and the use of only one prostate cancer cell line; these should be addressed in future research. Despite limitations related to model dependency and the experimental system, this study provides an important theoretical foundation for understanding the carcinogenic mechanisms of environmental pollutants and for developing targeted prevention and treatment strategies.

## Data Availability

The original contributions presented in the study are included in the article/Supplementary Material, further inquiries can be directed to the corresponding authors.
