# Supplementary material for: Deciphering the role of per- and polyfluoroalkyl substances in prostate cancer: a multi-omics and computational toxicology approach
Source: Front Cell Dev Biol. 2026 Jun 1;14:1786248. doi: 10.3389/fcell.2026.1786248 (PMC13265452; doi:10.3389/fcell.2026.1786248)
Supplement: Supplementary file 1 [file Supplementaryfile1.docx]

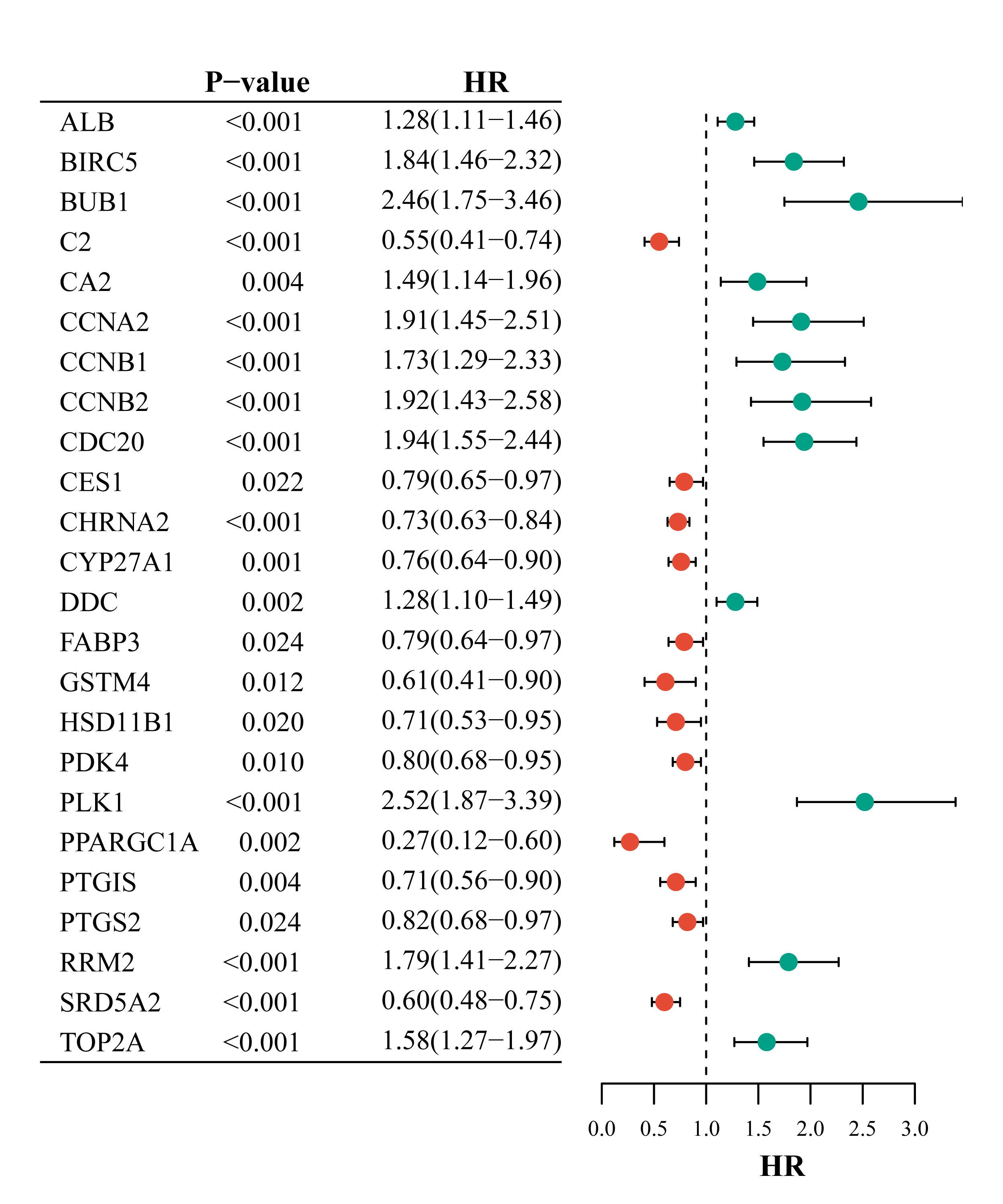


Figure S1. Forest Plot of COX Regression Results for the 24 Shared Prognostic Genes in the TCGA-PRAD Cohort


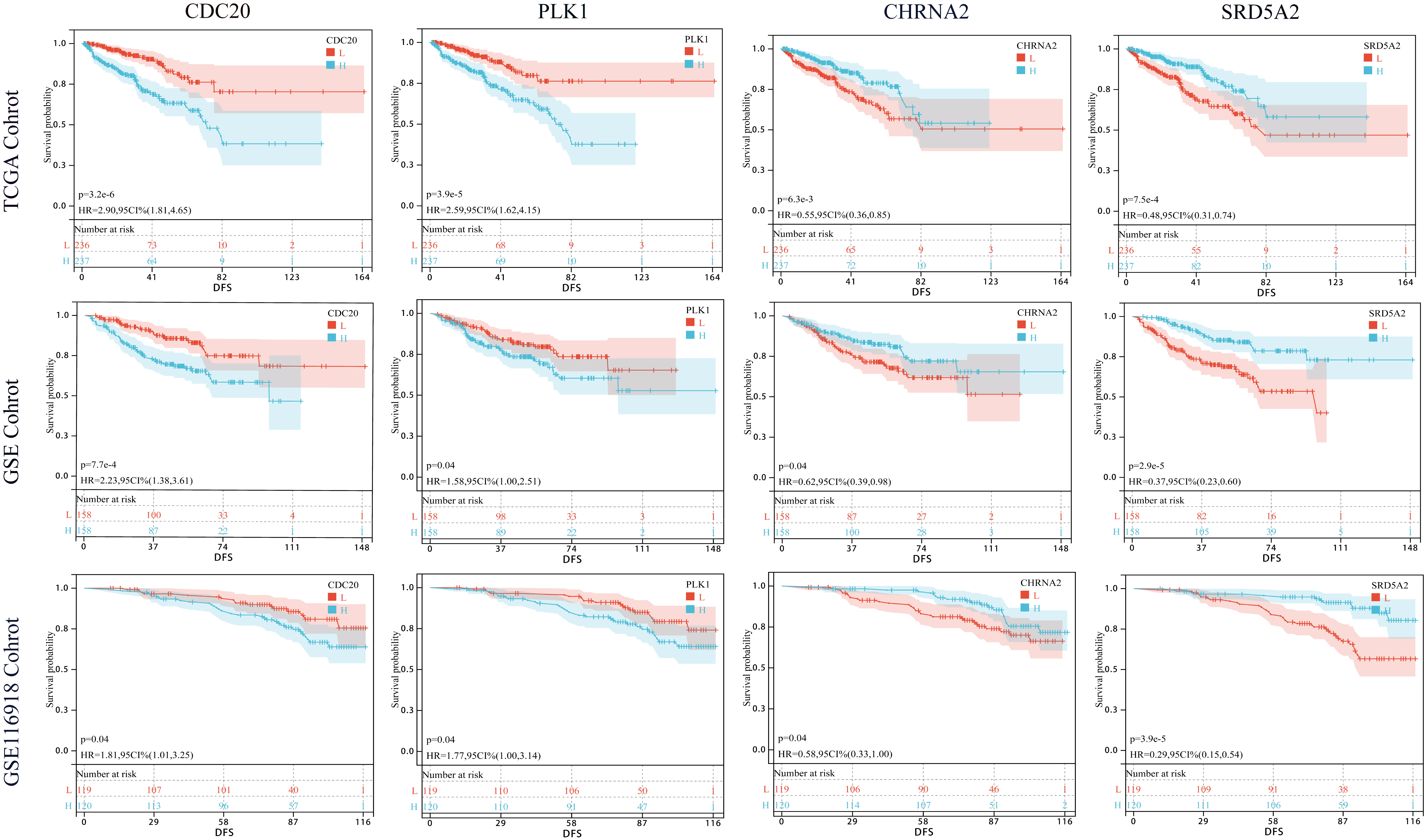


Figure S2. Prognostic Value of Core Genes: Kaplan-Meier Survival Stratification in Three Independent Cohorts


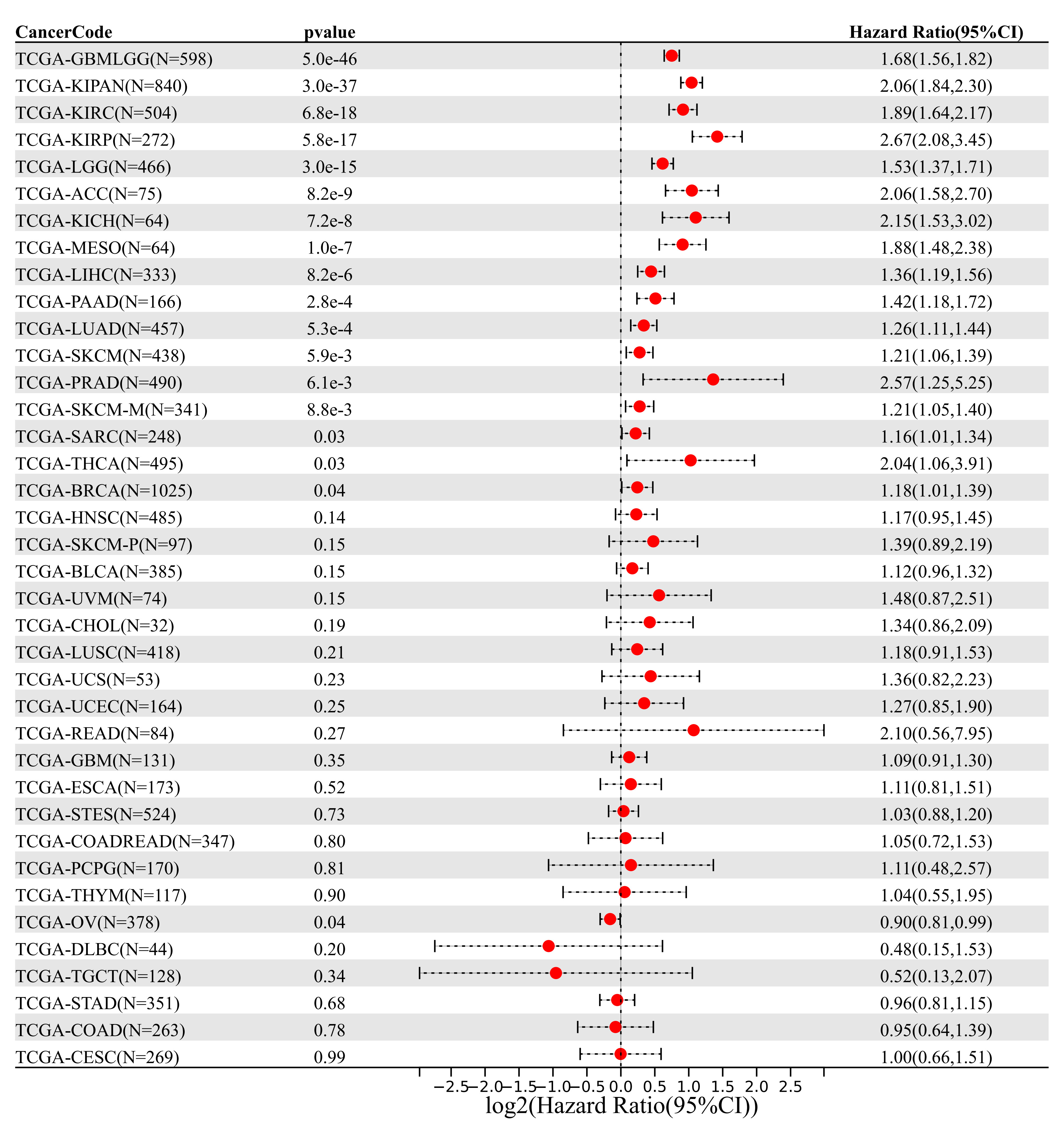


Figure S3. CDC20 and Disease-Specific Survival in Pan-Cancer Cohort


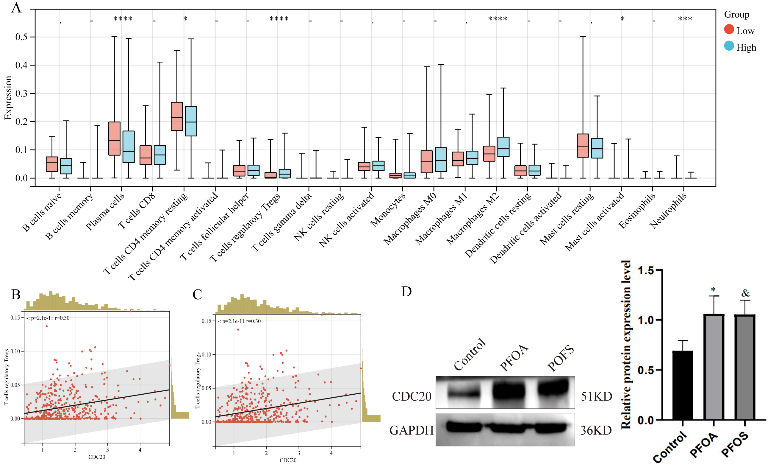


Figure S4. The impact of CDC20 on the tumor microenvironment. (A) Immune infiltration analysis showing higher infiltration levels of immunosuppressive cells (Tregs and M2 macrophages) in the CDC20 high‑expression group. (B, C) Correlation analysis confirming positive associations between CDC20 expression levels and the degree of immunosuppressive cell infiltration. (D) Western blot analysis showing that PFAS treatment (10 nM PFOA or PFOS, 48 h) significantly increases CDC20 protein expression in DU145 cells. Note: *p < 0.05 vs. control; &p < 0.05 vs. control.


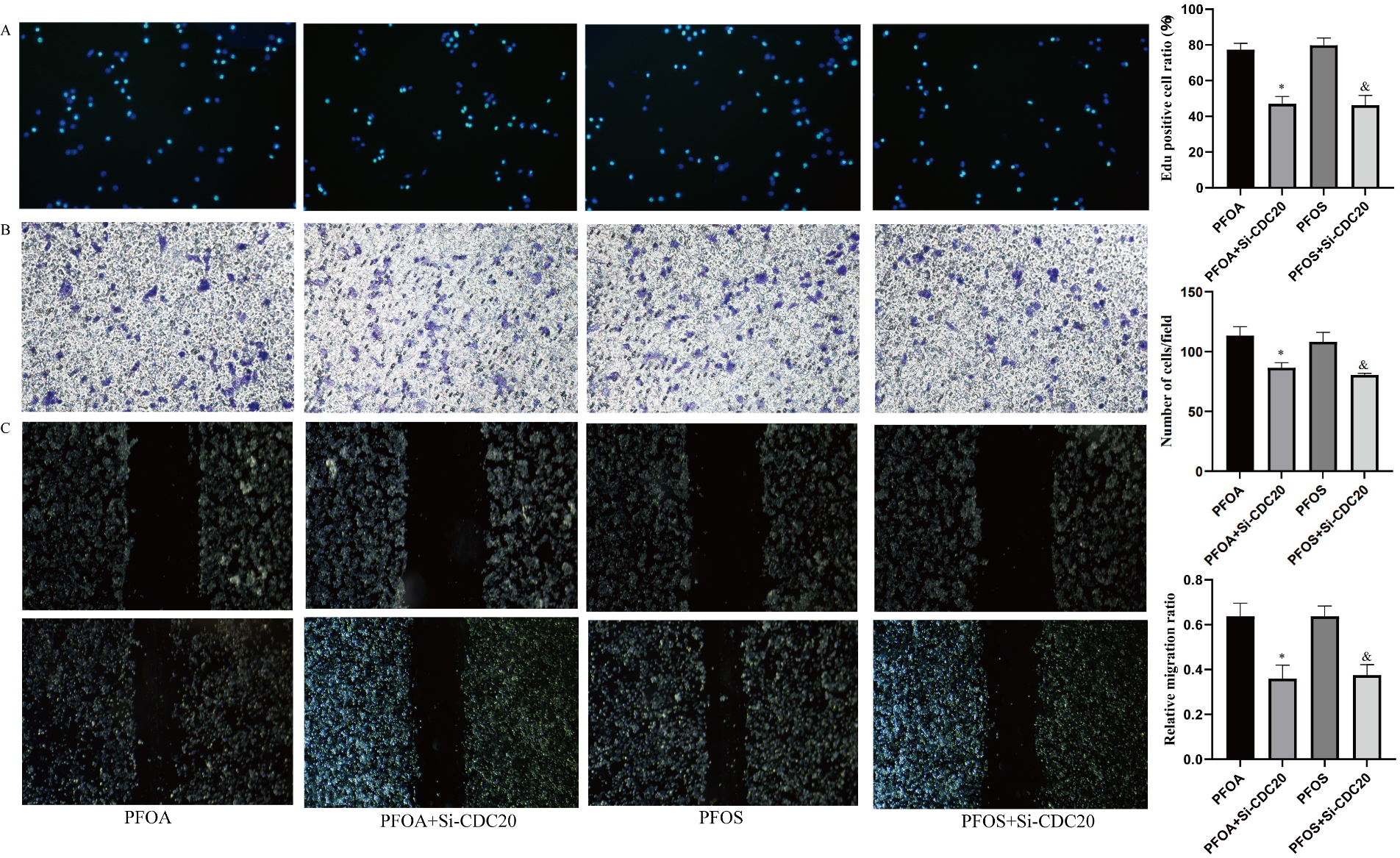


Figure S5. CDC20 knockdown attenuates PFAS‑induced migration in DU145 cells.

DU145 cells were treated with 10 nM PFOA or PFOS for 48 h, with or without prior transfection with CDC20‑specific siRNA (Si‑CDC20). Cell migration was assessed by wound healing assay. The relative migration ratio was calculated as the percentage of wound closure normalized to the control group (set as 1.0). Data are presented as mean ± SD. *p < 0.05 vs. PFOA; #p < 0.05 vs. PFOS. The results demonstrate that CDC20 knockdown significantly reverses the pro‑migratory effect of PFAS exposure.
